# The Relationship between Parental Rearing Behavior, Resilience, and Depressive Symptoms in Adolescents with Congenital Heart Disease

**DOI:** 10.3389/fcvm.2017.00055

**Published:** 2017-09-08

**Authors:** Ju Ryoung Moon, Jinyoung Song, June Huh, I-Seok Kang, Seung Woo Park, Sung-A Chang, Ji-Hyuk Yang, Tae-Gook Jun

**Affiliations:** ^1^Department of Nursing, Grown-Up Congenital Heart Clinic, Heart Vascular and Stroke Institute, Samsung Medical Center, Seoul, Korea; ^2^Department of Pediatrics, Grown-Up Congenital Heart Clinic, Heart Vascular and Stroke Institute, Samsung Medical Center, Sungkyunkwan University School of Medicine, Seoul, Korea; ^3^Division of Cardiology, Grown-Up Congenital Heart Clinic, Heart Vascular and Stroke Institute, Samsung Medical Center, Sungkyunkwan University School of Medicine, Seoul, Korea; ^4^Department of Thoracic & Cardiovascular Surgery, Grown-Up Congenital Heart Clinic, Heart Vascular and Stroke Institute, Samsung Medical Center, Sungkyunkwan University School of Medicine, Seoul, Korea

**Keywords:** parental rearing behavior, resilience, depression, congenital heart disease, adolescent

## Abstract

**Objectives:**

Parental rearing behavior is one factor that influences the strength of resilience. In turn, resilience influences depression. However, it is unclear whether resilience has a mediating effect on the relationship between parental rearing and depression in adolescents with congenital heart disease (CHD). Therefore, the associations between parental rearing behavior and resilience and between rearing behavior and symptoms of depression were investigated with respect to age, gender and disease severity.

**Subjects and methods:**

Patients completed a parental rearing behavior questionnaire, a resilience scale and the Children’s Depression Inventory during a routine clinic visit. Structural equation modeling with maximum likelihood estimation was used to analyze the data.

**Results:**

The median age of the 180 patients included in the study was 17.8 years, and 64% were male. Lower resilience was found to be associated with overprotection, punishment, rejection, and control. There was a strong relationship between resilience and symptoms of depression. Resilience varied according to gender, age group, and disease severity.

**Conclusion:**

Parental rearing behaviors such as emotional warmth, rejection, punishment, control, and overprotection have a significant influence on adolescent’s resilience. When developing intervention programs to increase resilience and reduce depression in adolescents with CHD, parenting attitudes, gender, age, and CHD severity should be considered.

## Introduction

Although outcomes have continued to improve following advances in cardiac surgical and catheter intervention, the negative impact of congenital heart disease (CHD) remains. Altered body image (from operation scars), interruptions to schooling, frequent hospital admissions, physical limitations, and parental overprotection of adolescents with CHD might contribute to psychosocial problems (1–3).

Depression is a serious health problem, especially in adolescence. Approximately 27–40% adolescents and adults with CHD are affected by depression (4). Previous studies reported that adolescents with CHD have more severe problems with depression and behavioral issues than healthy adolescents (2, 5). Depression, in adolescents with CHD, was reported to be significantly associated with “resilience, parental attitude, age, cyanosis, and school performance” (2).

Resilience refers to an individual’s ability to successfully adapt to life tasks in the face of social disadvantages or highly adverse circumstances (6). Individual levels of stress adaptation are determined by internal protective factors (e.g., optimism, perceptions of control, self-efficacy, and active coping) or external protective factors (e.g., social support system) (5). Thus, strengthening these protective factors is essential to facilitate adolescent healthy socio-psychological development (6–9).

In an earlier resilience study, parental rearing attitude was found to be a protective factor in adolescents (5). Also, positive associations were found between better mental health outcomes, high resilience, and supportive parenting (10, 11). Furthermore, Pereira et al. (7) and Swanson et al. (12) stated that the relationship between mental health outcomes and positive parenting rearing behavior was mediated by resilience. However, mediating effects of resilience between depression of adolescents with CHD and parental attitude were not examined.

Resilience interacts with developmental stages and changes and develops throughout the life course (12). Although Stratta et al. reported that there was a difference in resilience by gender (13), it is unclear how adolescents with CHD develop protective factors against the perceived risks by gender and age (14). Thus, a study about gender and age as moderators in the relationship between resilience and depression should provide foundational data about resilience in adolescents with CHD and intervention for their depression. In addition, because severity of disease is the major influencing factor for depression in adolescents with CHD (2, 14), it may be necessary to investigate how it affects resilience. Therefore, the aim of this study was to evaluate the mediating effect of resilience on parenting attitudes and depression, and to investigate whether gender, age, and severity of CHD affected the relationship between resilience and depression.

## Materials and Methods

### Materials and Procedures

This prospective study examined adolescents with CHD from an outpatient clinic, a single tertiary center. Inclusion criteria in this study were as follows: (1) 13–18 years old; (2) had a previous diagnosis of CHD and received cardiac surgery or intervention to correct a cardiac malformation; (3) had no history of intellectual handicap syndromes or complications(s) (e.g., trisomy 21); (4) were able to understand and answer the questionnaire, and (5) both the patient and their parents consented to take part in the survey. In total, 186 patients visited the CHD clinic, at the Samsung Medical Center during research periods. We excluded six patients, five of whom provided inadequate responses to the survey, and one who was diagnosed with Marfan syndrome. Thus, 180 patients were included in the final analysis. The sample size met the requirements for structural equation modeling (15–17).

The survey was conducted after the approval of the study protocol was obtained from the Samsung Medical Center Institutional Review Board. Once the patients and their parents agreed to participate, they signed a consent form. The patients completed a battery of questionnaires while awaiting their regular checkup appointments at the outpatient clinic. Responses from all patients were collected by one cardiovascular outpatient nurse.

### Instruments

Adolescents self-reported on standardized questionnaires designed to measure parental rearing behavior, resilience, depression, and general characteristics. The translation process for the parental rearing behavior and resilience instruments was based on Brislin’s translation model (18). After an initial translation into Korean by a bilingual medical doctor and a qualified bilingual expert, a blinded, qualified expert verified the meaning of each sentence using backward translation. An expert monolingual reviewer and a bilingual nursing professor then evaluated and modified the translation.

### Parental Rearing Behavior

An ultra-short screening version (US) of the Recalled Parental Rearing Behavior Questionnaire (19) (Fragebogen zum erinnerten elterlichen Erziehungsverhalten; FEE) (20, 21), was used to measure parental rearing behavior. The FEE-US is a shortened version of the Egna Minnen Betraffande Uppfostran (Own Memories of Child Rearing Experiences; EMBU) implemented by Petrowski et al. (19, 22) implemented by Petrowski et al. The FEE-US, utilizes scores on a four-point Likert scale (19), consists of 12 items (six items for each parent), and measures how often specific situations or circumstances were experienced by the participant (22). It has three scales: (1) paternal/maternal rejection and punishment, (2) paternal/maternal emotional warmth, and (3) paternal/maternal control and overprotection (23). The rejection and punishment scale assesses inappropriate behavior as perceived by a child, such as overly strictness and rejection (20, 22). The emotional warmth scale assesses behavior perceived positively from a respective parent, such as praise, support, and affection, without any unnecessary interference (22). Control and overprotection assess the following behaviors from a respective parent: overly thoughtful blaming, interfering, and constricting. These behaviors reflect the parents’ perspectives on performance, high expectations, and effort. The FEE-US score ranges from 2 to 8 and it is calculated by adding the value of each assigned item for all three scales, and for each parent (21). The psychometric properties of the three scales of the short version were found to be satisfactory to good (21). The Cronbach’s α in original version was 0.72–0.89 which indicated good reliability (24). Cronbach’s α in this study was 0.89.

### The Resilience Scale (RS)

Resilience was measured with a shortened version of Wagnild and Young’s RS (25), or the RS-11, as implemented by Schumacher et al. (26, 27). Resilience, as conceptualized by the RS, is defined as the ability to cope with development tasks by utilizing internal and external resources. The RS (original) was separated into two dimensions: (1) 17 items assessed personal competence and (2) 8 items assessed acceptance of self and life (25). Containment, persistence, independence, and self-value were assessed on the personal competence scale. Moreover, tolerance, flexibility, and adaptability were assessed on the acceptance of self and life scale (25). High scale values represented high resilience. Internal consistency on the original version reported by Schumacher et al. indicated very good reliability (Cronbach’s α = 0.91) (21, 26).

The RS-11 comprised items measured on a seven-point Likert scale (28), and reliability for this version correlated very highly with reliability for the original RS-25 version (*r* = 0.86) (28). Cronbach’s α in this study was 0.92. The RS items were simplified into two parcels. The two groups were organized by alternately assigning the items, from highest to lowest in factor loadings of the latent variable (21).

### Depression

The Korean version of the Children’s Depression Inventory (CDI) was developed by Cho and Lee (29, 30) based on Kovac’s modification of the Beck Depression Inventory for 8–18-year olds (31). The self-administered instrument is composed of 27 questions on patient feelings. Each item assessed depressive symptoms such as disturbed mood or apathy, in addition to school-related issues such as social rejection (31). Adolescents were asked to choose a sentence out of three, based on the severity of symptoms (no symptoms, mild symptoms, and severe symptoms) that they experienced during the past 2 weeks for each item. The score for each item ranged from 0 to 2 (2 represents the greatest symptom severity) and the total possible score ranges from 0 to 54 (a higher total score indicates a greater severity of depression). The CDI (Korean version) was found to be satisfactory with internal consistency (Cronbach’s α = 0.88) (29) and test–retest reliability (Cronbach’s α = 0.82) (29). Healthy adolescents had a mean score of 14.72 and scores between 11 and 13 represented the cutoff point for depression. In this study, Cronbach’s α was 0.82.

### Disease Severity

Congenital heart disease severity was measured by the Disease Severity Index (DSI). The DSI was developed to reflect the course of the illness (32, 33) and encompasses three levels of severity (low, moderate, and high). In this study, patients who received at most one cardiovascular surgery or one catheter intervention were considered low severity. The moderate severity group included patients who received more than one cardiovascular catheterization or intervention. Last, patients with persistent cyanosis were classified into the high severity group. These patients showed single-ventricle physiology or less than 92% oxygen saturation at rest (32, 33).

### Statistical Analysis

The data were analyzed using SPSS (version 22.0, IBM, Chicago, IL, USA) and AMOS (version 22.0, IBM, Chicago, IL, USA) software. Descriptive statistics were used to analyze demographic data, parental rearing behavior, resilience, and depression in adolescents with CHD. Independent sample *t*-tests, analysis of variance, and Scheffe tests were performed to identify differences in the level of parental rearing behavior, resilience, and depression according to age and disease severity. Prior to examining the goodness of fit of hypothesized model, validity examination among latent variables was conducted *via* the confirmatory factor analysis (CFA). The CFA examines the construction of observed variable, in which factor loading values above 1.96 is significant (15, 17). In this study, it is confirmed that each items of parental attitude, resilience, and depression are all factor loaded with values above 2.0. A structural equation modeling approach was used in order to evaluate the mediating effect of resilience. The χ^2^, degrees of freedom (df), goodness-of-fit index (GFI), normal fit index (NFI), comparative fit index (CFI), root mean square error of approximation (RMSEA), Tucker-Lewis index, and the parsimonious goodness-of-fit index were used in the goodness-of-fit tests for the model (15, 16, 34–36). Covariance matrices were used to test the model and the maximum likelihood method approach was used to estimate the model (15, 35).

Additionally, latent mean analysis (LMA) was performed to examine the difference of resilience by age, gender, and severity of disease (17, 37, 38). Although the difference between groups is often examined *via* the *t*-test or multivariate analysis of variance (MANOVA), these analyses may result in incorrect outcomes as they use measured variables that contain measurement errors. LAM controls for measurement errors to overcome the limitations of the *t*-test or MANOVA and to detect group differences in greater accuracy (37). To conduct LMA, the assumptions should be satisfied by the invariance test, that is, configural invariance, metric invariance, and scala invariance (37, 38). Configural invariance examines whether identical latent variables are loaded between groups so as to confirm identical basic structures. After that, metric invariance can be examined. It is to investigate whether factor coefficients are identical by controlling the identical factor loading among groups. Once this is confirmed, the next step is to test scalar invariance (38). Scalar invariance examination stipulates that respondents with identical values of latent variables should have identical observed values regardless of involved groups (38, 39). When examining the goodness of fit of a model *via* configural invariance, metric invariance, and scala invariance, fit indices should be as follows: CFI variance less than 0.01 and RMSEA variance less than 0.015 are regarded as identical models (34, 36). This study performs LMA to find differences in resilience by gender, age, and disease severity after examining the three processes (36). In LMA, a factor mean is not directly estimated, but through the differences between the averages of latent mean of the reference group and that of comparison group after controlling for the latent mean as zero. The interpretation on the latent mean difference is based on Cohen effect size (*d*). Cohen’s *d* is a value that divides a mean difference by the common SD. The reference group mean is compared with the mean of the comparison group on a standard score scale, which suggests the degrees of effectiveness. The *d* value = 0 indicates that the mean of the reference group and that of the comparison group are identical. The *d* value = 0.2 refers to a small effect size, 0.5 refers to a medium effect size, and 0.8 refers a large effect size (37).

## Results

**Figure 1:**
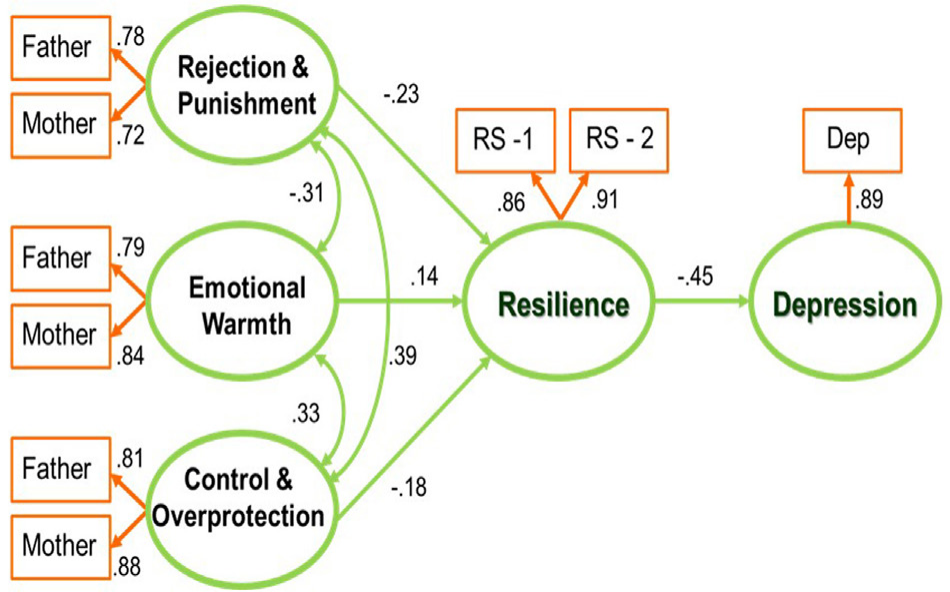
Path diagram for the hypothetical model. All coefficients are significant with *P* < 0.001.

Table 1 shows demographic and clinical data and Table 2 demonstrates descriptive statistics of the study variables. The first objective was to examine the relationship between depression, resilience, and parental rearing behavior. The hypothesized model described in Figure 1 is suitable for the data. Table 3 shows the total structural equation model and its fit indices. All of the fit indices satisfied the recommended levels (15, 17, 36).

**Table 1:** General and clinical characteristic of the subjects (*N* = 180).

Variable		Category	*N* (%)	Mean ± SD
Age (years)				15 ± 1.4
Gender		Male	115 (64.0)	
Religion		Yes	73 (40.6)	
Family structure		Extended	18 (10.0)	
		Nuclear	150 (83.3)	
		Single parent	12 (6.7)	
Academic achievement		High	53 (59.4)	
		Middle	100 (55.6)	
		Low	27 (15.0)	
Primary CHD diagnosis	Acyanotic CHD, 72 (40)	VSD	28 (15.0)	
		ASD	22 (12.3)	
		Valvar disease (TR, MR, AR)	15 (8.3)	
		CoA	7 (3.8)	
	Cyanotic CHD, 108 (60)	TOF	47 (26.1)	
		PA with VSD	19 (10.6)	
		Tricuspid A	10 (5.6)	
		DORV	15 (8.3)	
		TGA	6 (3.3)	
		TAPVR	4 (2.2)	
		HLHS	3 (1.7)	
		Truncus A	4 (2.2)	

*CHD, congenital heart disease; VSD, ventricular septal defect; ASD, atrial septal defect; TR, tricuspid regurgitation; MR, mitral regurgitation; AR, aortic regurgitation; CoA, coarctation of aorta; TOF, tetralolgy of fallot; PA, pulmonary atresia; Tricuspid A, tricuspid atresia; DORV, double outlet right ventricle; TGA, transposition of great arteries; TAPVR, total anomalous pulmonary venous return; HLHS, hypoplastic left heart syndrome; Truncus A, truncus arteriosus; SD, standard deviation*.

**Table 2:** Descriptive statistics for questionnaire of the subjects (*N* = 180).

Variables	All	Men	Women	*P*	Age		*P*	Disease severity			*P*
					13–15 years	16–18 years		Mild	Mod	Severe	
Emotional warmth (F)	4.31 (1.3)	3.85 (1.6)	4.53 (1.3)	<0.001	4.65 (1.2)	3.94 (1.3)	<0.001	4.45 (1.6)	4.19 (1.5)	3.94 (1.4)	<0.001
Emotional warmth (M)	5.21 (1.4)	5.03 (1.1)	5.50 (1.2)	0.009	5.67 (1.1)	5.04 (1.7)	<0.001	5.52 (1.4)	5.32 (1.4)	5.04 (1.2)	0.001
Control and overprotection (F)	3.13 (1.2)	3.08 (1.0)	3.15 (1.4)	0.301	3.32 (1.0)	2.89 (1.3)	0.014	3.37 (1.1)	32.7 (1.2)	3.12 (1.1)	0.011
Control and overprotection (M)	3.11 (1.3)	3.10 (1.1)	3.18 (1.5)	0.431	3.28 (1.4)	3.09 (1.1)	0.028	3.68 (1.4)	3.60 (1.3)	3.39 (1.0)	0.018
Rejection and punishment (F)	2.67 (1.1)	2.79 (1.1)	2.34 (1.0)	0.015	2.32 (1.2)	2.87 (1.2)	<0.001	2.62 (1.0)	2.75 (0.9)	2.87 (1.0)	0.022
Rejection and punishment (M)	2.87 (1.3)	2.73 (1.0)	2.92 (0.8)	0.154	2.75 (0.7)	2.97 (1.0)	0.032	2.43 (0.8)	2.5 (0.9)	2.67 (1.0)	0.021
Resilience	54.5 (10.6)	56.12 (9.2)	52.29 (10.0)	0.021	57.32 (11.0)	53.14 (9.7)	<0.001	60.42 (10.0)	58.49 (9.8)	55.51 (10.1)	<0.001
Depression	16.21 (5.6)	14.70 (6.2)	18.92 (5.9)	0.014	15.10 (6.3)	19.01 (4.9)	<0.001	13.12 (5.2)	16.32 (4.8)	18.21 (6.4)	<0.001

*F, father; M, mother; mod, moderate*.

*All data expressed as mean (SD)*.

**Table 3:** Test of the goodness of fit of the hypothetical model.

N	χ^2^ (df)	*P*	CMIN/DF	CFI	GFI	RMSEA	TLI	NFI	PNFI
180	25.73 (2)	<0.001	12.865	0.945	0.972	0.03	0.947	0.982	0.823

*df, degrees of freedom; CMIN/DF, minimum discrepancy, divided by its degree of freedom; CFI, comparative fit index; GFI, goodness-of-fit index; RMSEA, root mean square error of approximation; TLI, Tucker-Lewis index; NFI, normal fit index*.

All path coefficients with a *P*-value < 0.001 in the model are significant. The three dimensions of parental rearing behavior are weakly inter-correlated, as represented in Figure 1. Rejection and punishment (β = −0.23, *P* = < 0.001), emotional warmth (β = 0.14, *P* = 0.003), and control and overprotection (β = −0.18, *P* = 0.001) predict a level of resilience, which predicts depression (β = −0.40, *P* = < 0.001). For the three dimensions of parental rearing behavior, the standardized indirect effects on depression are small (range: −0.05 to 0.12). According to the results, we can confirm that resilience is a mediator between depressive symptoms and parental rearing behavior.

The equivalency of the model was tested, across gender, age, and severity groups, with additional analyses (34, 40). The multigroup analyses showed that configural invariances were larger than 0.90 of CFI and smaller than 0.50 of RMSEA, across gender, age, and disease severity. Also, metric invariances and scalar invariances were smaller than 0.01 of CFI and RMSEA (34, 40), across gender, age, and disease severity, as shown in Table 4. Configural invariance, metric invariance, and scala invariance were all examined. Therefore, the data suggest differences in resilience by gender, age, and disease severity with the structural equation model.

**Table 4:** Results of invariance across gender, age, and diseases severity (*N* = 180).

	N	χ^2^ (df)	*P* for Δχ^2^	CMIN/DF	CFI	ΔCFI	RMSEA	ΔRMSEA	Result
**Gender**									
Male	115	16.419 (2)		8.209	0.943			0.034	
Female	65	9.280 (2)		4.140	943			0.034	

**Multigroup analysis**									
Congfigural invariance		45.490 (4)		11.449	0.943		0.024		Accepted
Metric invariance		52.912 (6)	0.069	10.582	0.943	<0.001	0.029	0.001	Accepted
Scalar invariance		66.146 (9)	<0.001	7.349	0.959	0.012	0.028	0.001	Accepted

**Age group**									
13–15 years	95	13.579 (2)		6.789	0.952		0.069		
16–18 years	85	12.150 (2)		6.075	0.950		0.070		

**Multigroup analysis**									
Congfigural invariance		46.258 (10)		4.625	0.953		0.029	0.001	Accepted
Metric invariance		59.863 (10)	0.008	5.968	0.956	0.001	0.030	0.001	Accepted
Scalar invariance		64.891 (12)	0.002	5.407	0.951	0.004	0.031	0.001	Accepted

**Disease severity**									
Mild	62	8.862 (2)		4.431	0.936		0.030		
Moderate	83	11.850 (2)		5.925	0.945		0.031		
Severe	35	5.003 (2)		2.501	0.917		0.027		

**Multigroup analysis**									
Congfigural invariance		66.845 (14)	0.010	4.774	0.923		0.037	0.004	Accepted
Metric invariance		68.489 (16)	0.009	4.280	0.923	0.006	0.036	0.003	Accepted
Scalar invariance		89.321 (19)	0.004	4.701	0.952	0.003	0.038	0.001	Accepted

*df, degrees of freedom; CFI, comparative fit index; CMIN/DF, minimum discrepancy, divided by its degree of freedom; RMSEA, root mean square error of approximation*.

Table 5 examines group differences in resilience by gender, age, and disease severity. It suggests that boys as a reference group had a significantly lower latent mean than girls as a comparison group, and the effect size was as large as 0.96. When the age group of 13–15-year olds was used as a reference group, the age group of 16–18-year olds showed a significantly lower latent mean with the effect size of 0.89. Using the group with mild disease severity as a reference, the group with severe disease severity showed a significantly lower latent mean with the large effect size 1.36.

**Table 5:** Differences analysis of latent mean about variables.

Latent variables		Gender		Age		Disease severity		
		Boy	Girl	13–15 years	16–18 years	Mild	Moderate	Severe
Resilience	Latent mean	0	−0.69*	0	-0.75*	0	−0.26	−1.25*
	Mean	56.12	52.29	57.32	53.14	60.42	58.49	55.51
	Cohen’s *d*		0.96		0.89		0.35	1.36

*P < 0.001.

## Discussion

Resilience is defined as one’s ability to adapt successfully to adverse life circumstances, social disadvantages, and/or adversity (6). Many studies were conducted to understand the role of resilience in the development of depressive symptoms (7, 21). The results of this study verified that resilience has a mediating effect on parenting attitudes and depression. Moreover, an association was found between high resilience and positive parental rearing behavior (e.g., emotional warmth). In adolescents with CHD, depression may be explained by resilience and parenting attitudes, as shown in the multiple regression analysis. In addition, adolescents, who had an affectionate parent and high resilience, were found to be less depressed (2). This finding is consistent with a study in which children with chronic illnesses such as asthma had lower resilience scores and children whose parents were rigid and restrictive were more depressed (41). It is also partially consistent with the results of Pereira et al., which showed that both resilience and psychosocial functioning were predictors of depressive symptoms (7, 42).

A previous study of adolescents and adults found a relationship between parental rearing attitude, resilience, and psychological symptoms. Depressive symptoms and resilience were both negatively associated with negative parental rearing behaviors (21), which is consistent with the findings of the present study. Therefore, improving parenting attitudes can be a way to increase resilience and lessen depression. Furthermore, the harmful effects of negative parental rearing behavior on resilience may be corrected by “positive life experiences attained from social support systems, cohesion, networking, and relationships” (7, 21).

In this study, gender- and age-specific associations between parental rearing, resilience, and depression were found. The finding that resilience and the quality of experienced parental rearing differ according to gender is consistent with the results found by Stratta et al. (13). Boys showed slightly higher levels of resilience than girls in this study. This is partly consistent with Leppert et al.’s findings for adult subjects, that women had lower levels of resilience and worse physical symptoms than men, regardless of age (43). However, contrary to Leppert et al.’s findings, the present study found differences in the levels of resilience and depression according to age. In adolescence, parenting, resilience, and psychological symptoms may change and develop according to gender and age (11, 12). However, in adulthood, resilience as an intrapersonal resource decreases with age, while depression increases. There is also an argument that, due to reduced autonomy, protective strength of resilience decreases at this stage in life (44). Therefore, to understand resilience in adolescents with CHD, it is necessary to evaluate traits of resilience thoroughly, not only in adolescence, but also throughout the entire lifespan *via* longitudinal studies, and to apply the results to the development of interventions that increase resilience. Last, it was confirmed that the higher the severity of CHD, the lower the level of resilience. The results partially confirm the results of a previous study that suggested the CHD severity had a detrimental effect on resilience only if it was measured in poor functional status (33). Therefore, when developing an intervention program to increase resilience in adolescents with CHD and reduce depression, parenting attitudes, gender, age, and severity should be considered.

Intervention programs like “self-management training, art therapy, positive emotions, cognitive flexibilities, and social support” need to be developed in order to both prevent and decrease the risk of depressive symptoms in adolescents with CHD or any other chronic diseases (2). Moreover, these programs can help to bolster these adolescents’ skills in managing stress and increase their resilience (2, 44). These educational programs could provide information to parents on their roles in the development of their child or children. The goal of the information would be to promote a better understanding of CHD and to provide parents with the appropriate child-rearing methods, problem-solving, and communication skills in fostering their adolescents’ maturity (2, 14). These efforts and resources will help to develop adolescents’ resilience into adulthood and also educate them that their disease is manageable.

The authors previously examined resilience and parental attitude as major determinants of depression in adolescents with CHD (2). Based on the previous study, we were curious about depression, resilience, and parental attitudes, for which we examined their relationships. To the best of our knowledge, it is the first study in adolescent with CHD. We hope to see practical interventions provided after many follow-up studies conducted with patients with CHD.

This study has several limitations. First, the study’s sample is a convenient sample recruited from patients visiting a clinic for their regular checkups. Also, the results of the study may not be generalized as the sample was heterogeneous, including many patients in the moderate group of disease severity. Second, this study analyzed whether measured variables consistently represented the construct *via* CFA. Although this study used some promising screening tools, such as FEE-US and RS-11, they may not represent the full spectrum as retrospective assessment tools. Thus, we recommend replication studies with outcomes assessed by independent raters who are not family members, as observers. Additionally, DSI is a classification adopted in previous research (32, 33), which is not usual way adopted by cardiologists. Therefore, we recommend different classifications for follow-up studies as different results may come up based on various classifications.

## Conclusion

The strength of CHD adolescents’ resilience is significantly influenced by parental rearing behavior such as control, overprotection, rejection, punishment, and affection. The effect of resilience depends on gender and has varying effects according to age and disease severity. Therefore, when developing an intervention program to increase resilience of adolescents with CHD and reduce depression, parenting attitudes, gender, age, and severity should be considered.

## Ethics Statement

All of the participants gave their written informed consent, and the protocol of the study was approved by the Institutional Review Board at Samsung Medical Center.

## Author Contributions

Study conceived and co-designed by J. Paper drafted by JR, who also participated in the design and coordination studies and conducted statistical analyses. JS and I-SK assisted with design and manuscript drafting. SJ and SP helped to interpret the data as well as assisted in drafting the manuscript. JY, T-GJ, and J all helped to design the study and edited the vital content. The final manuscript was read and approved by all authors.

## Conflict of Interest Statement

The authors declare that the research was conducted in the absence of any commercial or financial relationships that could be construed as a potential conflict of interest.

## References

[1] Alden B Gilljam T Gillberg C. Long-term psychological outcome of children after surgery for transposition of the great arteries. Acta Paediatr (1998) 87:405–10. 10.1111/j.1651-2227.1998.tb01468.x 9628296

[2] Moon JR Huh J Kang IS Park SW Jun TG Lee HJ. Factors influencing depression in adolescents with congenital heart disease. Heart Lung (2009) 38:419–26. 10.1016/j.hrtlng.2008.11.005 19755192

[3] Meuleners LB Lee AH Binns CW Lower A. Quality of life for adolescents: assessing measurement properties using structural equation modelling. Qual Life Res (2003) 12:283–90. 10.1023/A:1023221913292 12769141

[4] Wang Q Hay M Clarke D Menahem S. The prevalence and predictors of anxiety and depression in adolescents with heart disease. J Pediatr (2012) 161:943–6. 10.1016/j.jpeds.2012.04.010 22640871

[5] Lee TY Cheung CK Kwon WM Resilience as a positive youth development construct: a conceptual review. Sci World J (2012) 2012:390450 10.1100/2012/390450 PMC3353472 22623893

[6] Pecillo M. The concept of resilience in OSH management: a review of approaches. Int J Occup Saf Ergon (2016) 22:291–300. 10.1080/10803548.2015.1126142 26652938 PMC4867880

[7] Pereira L Matos AP Pinheiro MDR Costa JJ Resilience and Depressive Symptomatology in Adolescents: The Moderator Effect of Psychosocial Functioning. Future Academy (2015). Available from: http://www.futureacademy.org.uk/files/images/upload/7ichandhpsy2016.pdf

[8] Masten AS. Resilience in developing systems: progress and promise as the fourth wave rises. Dev Psychopathol (2007) 19:921–30. 10.1017/S0954579407000442 17705908

[9] Zolkoski S Bullock L Resilience in children and youth: a review. Child Youth Serv Rev (2012) 34:2295–303. 10.1016/j.childyouth.2012.08.009

[10] Bandura A Self-Efficacy: The Exercise of Control. New York: Freeman (1997).

[11] Kobasa SC Maddi SR Kahn S Hardiness and health: a prospective study. J Pers Soc Psychol (1982) 42:168–77. 10.1037/0022-3514.42.1.168 7057354

[12] Swanson J Valiente C Lemery-Chalfant K O’Brien TC Predicting early adolescents’ academic achievement, social competence, and physical health from parenting, ego resilience, and engagement coping. J Early Adolesc (2011) 31:548–76. 10.1177/0272431610366249

[13] Stratta P Capanna C Patriarca S de Cataldo S Bonanni RL Riccardi I Resilience in adolescence: gender differences two years after the earthquake of L’Aquila. Pers Individ Dif (2013) 54:327–31. 10.1016/j.paid.2012.09.016

[14] Moon JR A Model of Quality of Life in Adolescents with Congenital Heart Disease [Dissertation]. Seoul: Catholic University (2005).

[15] Woo JP The Concept and Understanding of Structural Equation Modeling with AMOS 4.0–20.0. Seoul: Hanna Rae (2012).

[16] Moon JR Cho YA Huh J Kang IS Kim DK Health Qual Life Outcomes (2016). 10.1186/s12955-016-0488-5 PMC4890270 27249938

[17] Bae BR Structural Equation Modeling with AMOS 17.0. Seoul: Chungram (2009).

[18] Brislin RW Back-translation for cross-cultural research. J Cross Cult Psychol (1970) 1:185–216. 10.1177/135910457000100301

[19] Petrowski K Paul S Zenger M Brahler E. An ultra-short screening version of the Recalled Parental Rearing Behavior questionnaire (FEE-US) and its factor structure in a representative German sample. BMC Med Res Methodol (2012) 12:169. 10.1186/1471-2288-12-169 23134704 PMC3534221

[20] Edel M Juckel G Brune M Interaction of recalled parental ADHD symptoms and rearing behavior with current attachment and emotional dysfunction in adults offsping with ADHD. Psychiatry Res (2010) 178:137–41. 10.1016/j.psychres.2010.04.004 20452044

[21] Petrowski K Brahler E Zenger M The relationship of parental rearing behavior and resilience as well as psychological symptoms in a representative sample. Health Qual Life Outcomes (2014) 12:95 10.1186/1477-7525-12-95 25381113 PMC4289338

[22] Petrowski K Berth H Schmidt S Schumacher J Hinz A Brahler E. The assessment of recalled parental rearing behavior and its relationship to life satisfaction and interpersonal problems: a general population study. BMC Med Res Methodol (2009) 9:17. 10.1186/1471-2288-9-17 19267894 PMC2674060

[23] Osborne TL Jensen MP Ehde DM Hanley MA Kraftt G Psychosocial factors associated with pain intensity, pain-related interference, and psychological functioning in persons with multiple sclerosis and pain. Pain (2007) 127:52–62. 10.1016/j.pain.2006.07.017 16950570

[24] Arrindell WA Sanavio E Aguilar G Sica C Hatzichristou C Eisemann M The development of a short version of the EMBU: its appraisal with students in Greece, Guatemala, Hungary and Italy. Pers Individ Diff (1999) 27:613–8. 10.1016/S0191-8869(98)00192-5

[25] Wagnild GM Young HM. Development and psychometric evaluation of the Resilience Scale. J Nurs Meas (1993) 1:165–78. 7850498

[26] Schumacher J Leppert K Gunzelmann T Strauß B Brähler E Die Resilienzskala–Ein Fragebogen zur Erfassung der psychischen Widerstandsfähigkeit als Personmerkmal. Z Klin Psychol Psychiatr Psychother (2005) 53:1–92.

[27] Kocalevent RD Zenger M Heinen I Dwinger S Decker O Brahler E. Resilience in the general population: standardization of the resilience scale (RS-11). PLoS One (2015) 10:e0140322. 10.1371/journal.pone.0140322 26523927 PMC4629910

[28] Rausch S Herzog J Thome J Ludascher P Muller-Engelman M Steil R Women with exposure to childhood interpersonal violence without psychiatric diagnoses show no signs of impairment in general functioning, quality of life and sexuality. Borderline Personal Disord Emot Dysregul (2016) 3:13 10.1186/s40479-016-0048-y 27761262 PMC5055655

[29] Cho SC Lee YS Development of Korean from of Kovacs children depression inventory. J Korean Neruopsychiatr Assoc (1990) 29:943–56.

[30] Park S Lee J Baik YB Kim K Yun HJ Kwon H A preliminary study of the effects of an arts education program on executive function, behavior and brain structure in a sample of nonclinical school aged children. J Child Neurol (2015) 30:1757–66. 10.1177/0883073815579710 25862737

[31] Beck AT Depression: Clinical, Experimental and Theoretical Aspect. New York: Harper & Row (1967).

[32] Miller MR Forrest CB Kan JS Parental preferences for primary and specialty care collaboration in the collaboration in the management of teenagers with congenital heart disease. Pediatrics (2000) 106:264–9. 10.1542/peds.106.2.264 10920149

[33] Bang JS Jo S Kim GB Kwon BS Bae EJ Noh CI The mental health and quality of life of adult patients with congenital heart disease. Int J Cardiol (2013) 170:49–53. 10.1016/j.ijcard.2013.10.003 24139784

[34] Byrne BM Testing for multi-group invariance using AMOS graphics: a road less travelled. Struct Equ Modeling (2004) 11:272–300. 10.1207/s15328007sem1102_8

[35] Arbuckle JL AMOS TM 18 User’s Guide. Chicago: SPSS Inc. (2009).

[36] Cheung GW Rensvold RB Evaluating goodness-of-fit indexes for testing measurement invariance. Struct Equ Modeling (2002) 9:233–55. 10.1207/S15328007SEM0902_5

[37] Cole DA Maxwell SE Arvey R Salas E Multivariate group comparisons of variables systems: MANOVA and structural equation modeling. Psychol Bull (1993) 114:174–84. 10.1037/0033-2909.114.1.174

[38] Park KM Han AE Cho YH Construct equivalence and latent means analysis of health behaviors between male and female middle school students. Asian Nurs Res (2011) 5:216–21. 10.1016/j.anr.2011.12.002 25030523

[39] Chen FF Sensitivity of goodness of fit indices to lack of measurement invariance. Struct Equ Modeling (2007) 14:467–504. 10.1080/10705510701301834

[40] Lau W Hui CH Lam J Lau E Cheung S The relationship between spirituality and quality of life among university students: an autoregressive cross-lagged panel analysis. High Educ (2016) 69: 977–90. 10.1007/s10734-014-9817-y

[41] Kim DH Yoo IY. Factors associated with depression and resilience in asthmatic children. J Asthma (2009) 44:423–7. 10.1080/02770900701421823 17654126

[42] Miller-Lewis LR Searle AK Sawyer MG Baghurst PA Hedley D. Resource factors for mental health resilience in early childhood: an analysis with multiple methodologies. Child Adolesc Psychiatry Ment Health (2013) 7:6. 10.1186/1753-2000-7-6 23432929 PMC3598384

[43] Leppert K Strauß B Die Rolle von Resilienz für die Bewältigung von, Belastungen im Kontext von Altersübergängen. Z Gerontol Geriatr (2011) 44:313–7. 10.1007/s00391-011-0193-2 21892673

[44] Richter-Kornweitz A Gleichheit und Differenz–die Relation zwischen Resilienz, Geschlecht und Gesundheit. In: Wiesbaden ZM, editor. Handbuch Resilienzförderung. Wiesbaden: VS Verlag (2011). p. 240–74.

